# Preparation and properties of phase-change materials with enhanced radial thermal conductivities based on anisotropic graphene aerogels

**DOI:** 10.1039/d3ra06835h

**Published:** 2024-01-16

**Authors:** Jinhui Huang, Xuejiao Sun, Bing Liang, Ziyao Li, Danyang Zheng, Banglong Yang, Jiatao Xu, Yongchuang Zhu

**Affiliations:** a School of Chemical Engineering and Technology, Guangdong Industry Polytechnic Guangzhou 510330 China 2016103060@gdip.edu.cn +86-20-61230200; b Guangdong Engineering Technical Research Center for Green Household Chemicals Guangzhou 510330 China

## Abstract

In this study, anisotropic graphene aerogels are prepared using the heat-flow method. Then, graphene aerogels with nanosilver particles are prepared *via* a silver mirror reaction. The aerogels are soaked in paraffin wax and the effects on the properties of the wax are investigated. The thermal conductivity of pure paraffin wax is 0.2553 W m^−1^ K^−1^. For the prepared PCM, the aerogel content was 0.92 vol%; this increases to 1.2234 W m^−1^ K^−1^, which corresponds to a thermal conductivity enhancement efficiency of 582%. The axial thermal conductivity is 1.4953 W m^−1^ K^−1^, which corresponds to a thermal conductivity enhancement efficiency of 746%. The graphene aerogels with the nanosilver particles show high phase-change efficiency. Owing to the significant improvements in the axial and thermal conductivities, the radial and axial heat transfer properties show good consistency suitable for practical applications.

## Abstract 2

In this study, anisotropic graphene aerogels are prepared using the heat-flow method.

## Graphical abstract

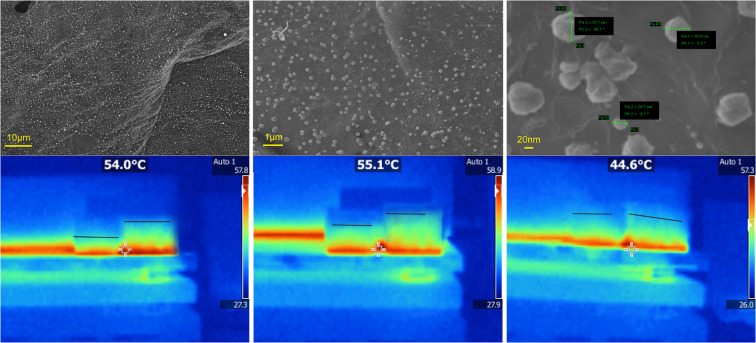

## 1. Introduction

Energy is vital for economic development and social progress. Historically, energy supplies have depended on non-renewable resources such as coal, oil, and natural gas, which are of limited supply and negatively affect the environment.^1^ Therefore, researchers have begun to explore renewable and sustainable energy resources. To ensure that the energy supply is reliable and to prevent energy shortages, it is necessary to develop methods of storing the energy obtained from renewable resources. Among the proposed energy storage technologies, thermal energy storage technologies such as latent heat storage (phase-change energy storage) have attracted particular attention.^2^ Phase-change energy storage uses phase-change materials (PCMs) with special functions that change the phase state at a given temperature or temperature range (phase-change temperature). As these materials change state, they absorb or release a large amount of latent heat, which can be used to store energy. In contrast to sensible heat storage and chemical energy storage technologies, phase-change energy storage does not involve large temperature variations and it is simple, convenient, and adaptable. Therefore, it is promising for practical applications and has great significance for the storage, recovery, and reuse of renewable energy.^3^

Organic medium- and low-temperature PCMs including polyvinyl alcohol, paraffin wax, and fatty acids are commonly used for applications such as solar energy collection,^4^ room temperature control, and heat dissipation from electronic components owing to their variety, low corrosivity, and mild operating conditions. However, they face some challenges, such as low thermal conductivity, poor thermal stability, flammability, undercooling, and molten-phase leakage,^5^ which prevent their widespread application. Through scientific studies and practical applications, it has been shown that organic PCMs can improve the utilization efficiency of heat energy.^1,2^ However, their low thermal conductivities and molten-phase leakage are serious problems that must be addressed. Researchers have made significant efforts to improve the thermal conductivities of organic PCMs and to develop effective packaging methods. In recent years, three-dimensional (3D) porous multifunctional materials, such as polyethylene, metal foams, ethylene glycol, and carbon aerogels, have been used to produce thermally conductive pathways and support networks to improve the thermal conductivity and shape stability of organic PCMs.

Graphene has attracted the attention of researchers owing to its ultrahigh thermal conductivity,^6,7^ ultrahigh specific surface area, and low density. It can be used as a thermally conductive filler to significantly improve the thermal conductivity of organic PCMs without affecting their latent heat values. However, the effect is dependent on the geometric structure of the graphene sheets including the layer number, size, and aspect ratio. Researchers have also considered 3D structural materials with anisotropic network structures for use as heat conduction paths.^8^ By controlling the microstructure, two-dimensional materials can be assembled into 3D materials and arranged in parallel contact in a certain direction, rather than being arranged in angular or vertical contact.^9^ Thus, the thermal conductivity in the direction parallel to this arrangement is greatly improved and the difference between the conductivity in the radial and axial direction is evident. The efficiency of a network with anisotropic thermal conductivity is significantly higher than that of a network with isotropic thermal conductivity.

Conrado *et al.*^10^ prepared isotropic and anisotropic aerogels using direct hydrothermal and ice-templating methods, respectively, and used them to enhance the thermal conductivity of epoxy resins. The anisotropic aerogels had higher thermal conductivity enhancement efficiencies. Li *et al.*^11^ also prepared graphene aerogels using the ice-templating method. After epoxy resin absorption, they had a thermal conductivity of 6.57 W m^−1^ K^−1^. Yang *et al.*^12^ used an ice-templating method with graphene oxide and boron nitride to prepare composite aerogels by adjusting the freezing temperature and distance from liquid nitrogen. After PEG adsorption, the thermal conductivity reached 3.18 W m^−1^ K^−1^.

Although anisotropic graphene networks can significantly improve the thermal conductivity of PCMs in the axial direction, the performance in the radial direction is still insufficient.^11^ Therefore, some researchers have attempted to connect materials with high thermal conductivity to graphene sheets in the axial direction to improve the radial thermal conductivity and phase-change efficiency. For example, composites consisting of graphene nanosheets decorated with silver nanoparticles combined with polyethylene glycol (Ag-GNS/PEG) have been used to collect visible light and convert it into heat energy (*η* = 88.7–92.0%).^13,14^ These Ag-GNS/PEG composites also have high energy storage capacities (166.1–177.2 J g^−1^), thermal conductivities (49.5–95.3%), thermal energy storage/release rates, and shape stabilities. Radhakrishnan *et al.*^15^ prepared hybrid graphene nanoparticle (GNP)–Ag nanoparticles using an *in situ* reduction method. When 1 wt% of hybrid GNP–Ag nanoparticles was added to the basic PCM, the effective thermal conductivity increased by 52%. Kalidasan *et al.*^16^ demonstrated that when the mass fraction of graphene (Gr):Ag nanopowder was 0.8 (RT50-0.8Gr:Ag), the thermal conductivity of paraffin wax (RT50) increased by 53.85%. However, materials with sufficiently high axial and radial thermal conductivities are still required to improve the prospects of PCMs for practical applications.

Therefore, this study aims to prepare a PCM with high axial and radial thermal conductivities by preparing anisotropic graphene aerogels *via* a heat-flow method and then attaching nanosilver particles to the walls of the aerogels. This will greatly improve the overall thermal conductivities of PCMs and can be used for many practical applications.

## 2. Materials and methods

### 2.1. Preparation of anisotropic graphene aerogels

**Fig. 1:**
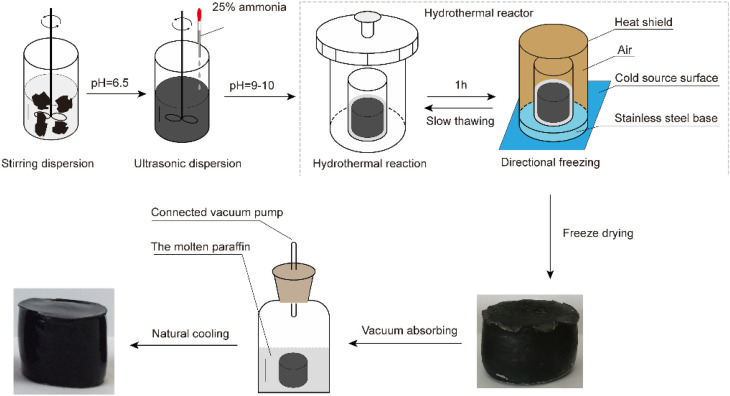
Process used to prepare the anisotropic graphene aerogels.

The process used to prepare the anisotropic graphene aerogels is shown in Fig. 1. First, 0.5 g of graphene oxide (GO; self-made) was added to a glass bottle filled with 50 mL of water and dispersed by stirring. The pH was adjusted to 6.5 ± 0.2 using ammonia water. Then, the mixture was sonicated at 10 °C with circulating water for 1.5 h. Next, the pH was adjusted to 9–10 using ∼25% concentrated ammonia water and placed in an oven at 90 °C for 1 h to facilitate the hydrothermal reaction. The sample was removed from the over, cooled *via* directional freezing, thawed at room temperature (∼25 °C), and placed back in the other at 90 °C for 3 h. When the sample was removed from the oven again, it was cooled *via* directional freezing at −45 °C for 3 h and then freeze-dried for 48 h. Thus, reduced GO aerogels (RGAs) were obtained. The RGAs were immersed in molten paraffin wax (Guangzhou Reagent #52) for 30 min under a vacuum (∼50 Pa) and then left to cool naturally. Finally, the RGA/paraffin composite PCMs (PRGAs) were obtained.

### 2.2. Preparation of Ag–graphene aerogels and their composite PCMs

**Fig. 2:**
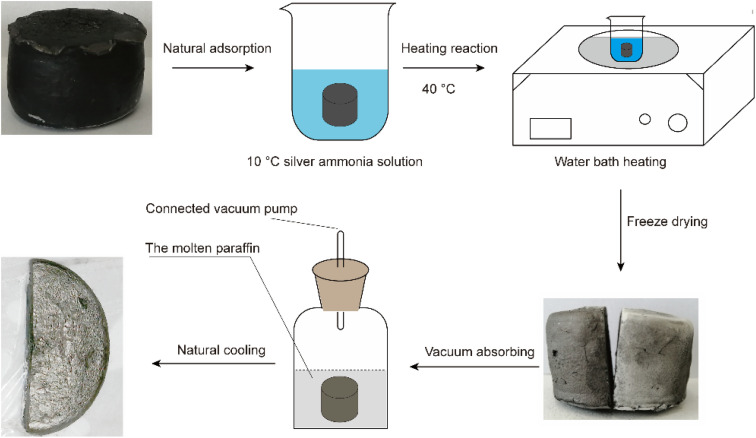
Process used to prepare the Ag–graphene aerogels.

The process used to prepare the Ag–graphene aerogels is shown in Fig. 2. First, a 2% silver ammonia solution was prepared and cooled to approximately 10 °C. Then, an appropriate amount of acetaldehyde solution was added. The RGA prepared in Section 2.1 was immersed in the solution, saturated *via* vacuum adsorption, and then placed on a heating plate at 40 °C for 1 h. The resulting silver-attached graphene wet gel was then freeze-dried to obtain the desired silver-attached graphene aerogel (RGA2A). The aerogel was immersed in molten paraffin wax (Guangzhou Reagent #52) for 30 min under a vacuum (∼50 Pa) and left to cool naturally. Finally, the RGA2A2/paraffin composite PCMs (PRGA2As) were obtained. The procedure was repeated with a 6% silver ammonia solution to obtain graphene aerogels (RGA6A) and their paraffin composite PCMs (PRGA6As).

### 2.3. Performance and structural characterization

A Carl Zeiss EVO18 microscope (SEM, Carl Zeiss, Jena, Germany) was used to observe the surface morphology of samples. Energy-dispersive X-ray spectroscopy (EDS, EVO18) was used to analyze the elemental distribution of the sample. A differential scanning calorimetry (DSC, Q2000, TA Instruments, New Castle, DE, USA) was used to explore phase change temperatures and phase change enthalpies of samples at a heating/cooling rate of 10 °C min^−1^ in a highly purified nitrogen atmosphere. The thermal conductivity of samples was measured using a thermal conductivity meter (hot-wire method, TC3100, XIATECH, China) at room temperature (*ca.* 25 °C). A data acquisition unit (2638A HYDRA SERIES III, Fluke, USA) with a heat flow sensor (HS-30, Captec, Villeneuve Dascq, France) were used for investigating the heat transfer behavior of samples. The data acquisition unit with probe and test lead (TL71, Fluke, USA) also was used for testing current.

The enhancement efficiency in thermal conductivity of the paraffin composites can be calculated by*η* = (*K* − *K*_m_)/(100 × *V* × *K*_m_) × 100%where *η* is the thermal conductivity enhancement efficiency; *K*_m_ and *K* are the thermal conductivities of paraffin and its composite, respectively; and *V* is the volume content of AGA in the composite.

## 3. Results

### 3.1. Structural characteristics of the samples

**Fig. 3:**
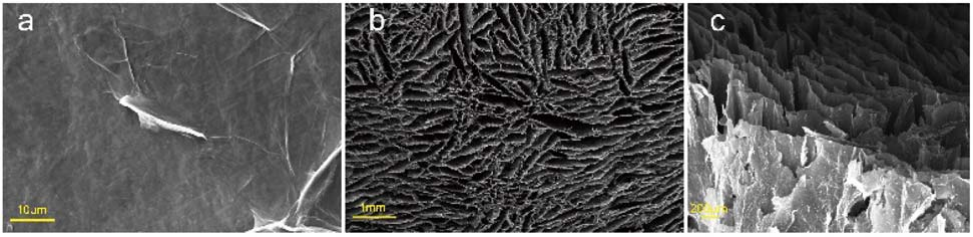
SEM images of (a) GO and (b) cross-section and (c) longitudinal section of the anisotropic aerogel.

Scanning electron microscope (SEM) images revealed that the samples had oriented structures, as shown in Fig. 3. The cross-section (Fig. 3b) showed many long holes of approximately the same size. The longitudinal section (Fig. 3c) showed that the GO sheets which were large sheets and could be spread flat in an aqueous solution (Fig. 3a) were arranged along the axial direction, forming the walls of the holes. This structure formed because a unidirectional cold source was used during the freezing process, which caused the ice crystals to grow slowly upward from the bottom cold source. The GO sheets were arranged along the ice crystal plane. After hydrothermal reduction, the GO sheets were connected and laminated to form sheet-like pore walls and graphene aerogels with evident anisotropy.

**Fig. 4:**
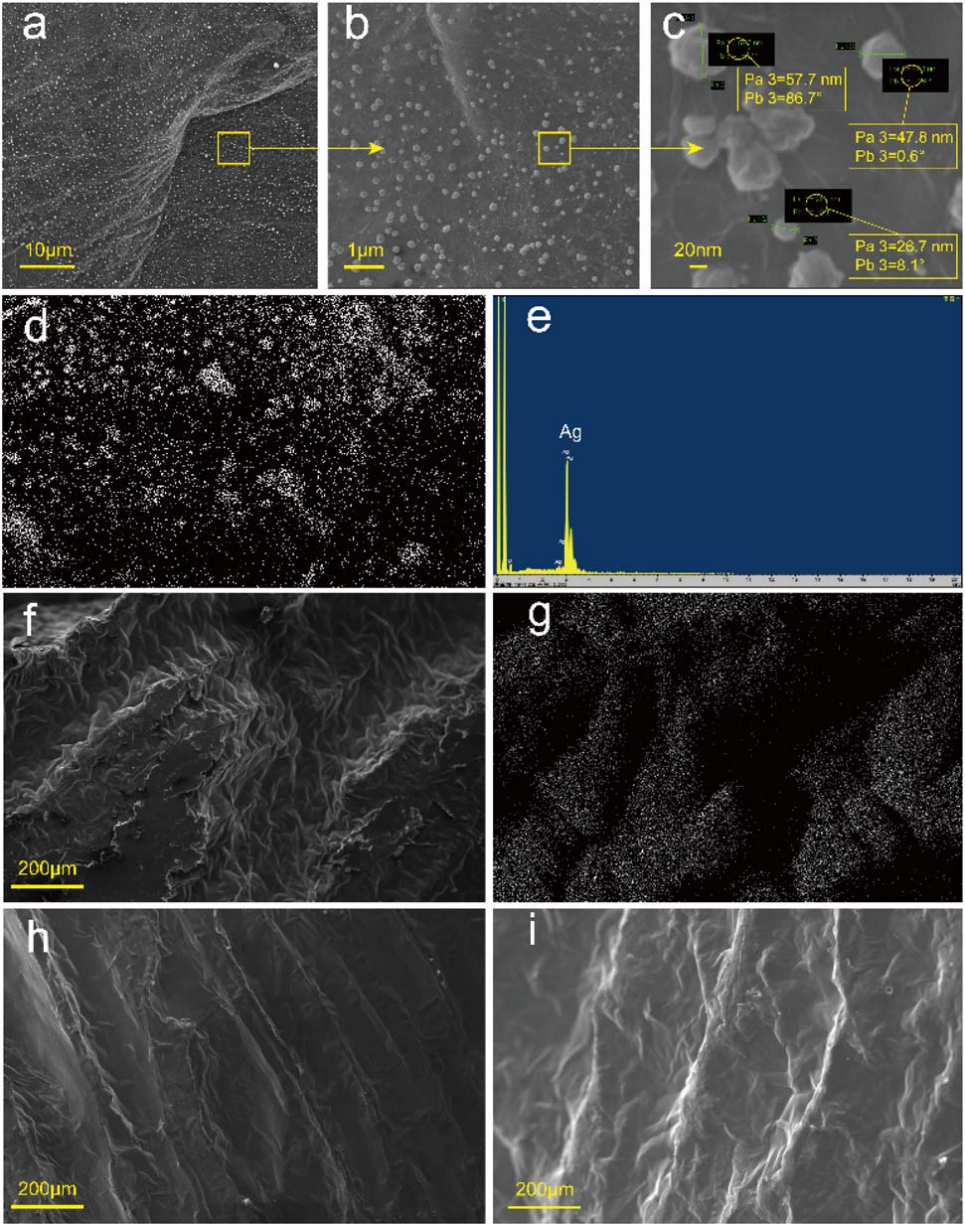
SEM and EDS diagrams of samples ((a)–(c) are different magnification electron microscope diagrams of RGA6A, (d) is EDS scanning diagrams of RGA6A, (e) is EDS elemental analysis diagrams of RGA6A, (f) is SEM diagrams of PRGA6A, (g) is EDS diagrams of PRGA6A, (h) is SEM diagrams of PRGA2A, and (i) is SEM diagrams of PRGA).

The RGA was used to prepare RGA6A and PRGA6A according to the method in Section 2.2 and the microstructures of both samples were observed. As shown in Fig. 4a–c, a large number of particles ∼120 nm in diameter were uniformly attached to the graphene sheets. Fig. 4d and e show that the attached particles were composed of silver and the silver content of the material was 4.3%. This shows that the silver particles were successfully attached to the graphene sheets *via* the silver ammonia solution reduction method. Because the silver particles were produced by solution reduction, their size was effectively controlled by the temperature. The formation process produced good contact with the graphene at the atomic level, which resulted in a good attachment effect.

After adsorbing the paraffin, the samples surface showed a gully-like structure (Fig. 4f, h and i) as the paraffin shrunk as it solidified with the greatest shrinkage in the middle of the holes. Energy dispersive spectrometry (EDS) (Fig. 4g) showed that there were many silver particles along the hole walls. However, there were almost no silver particles in the gullies because the holes were almost entirely filled with paraffin. Wide bands of silver particles appeared near the holes owing to the deformation of the hole walls caused by the solidification and shrinkage of the paraffin.

### 3.2. Thermal performance analysis

**Fig. 5:**
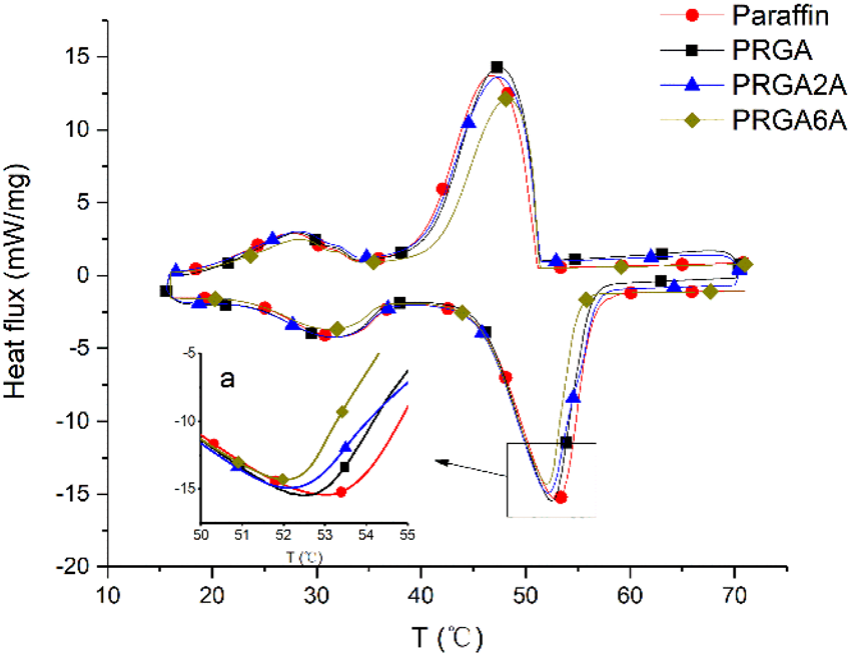
DSC curves for the various samples.

**Fig. 6:**
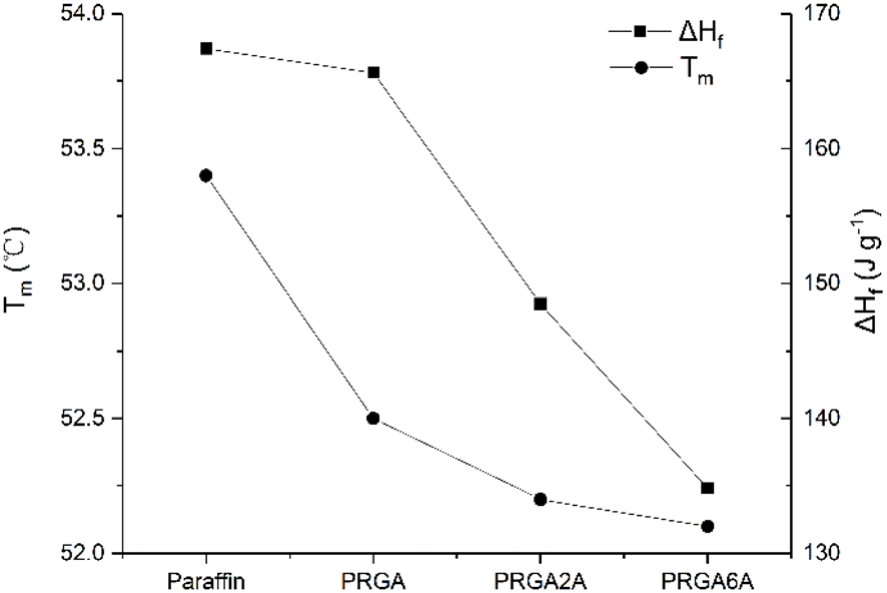
Curves of melting point and latent heat of fusion of samples.

Differential scanning calorimetry (DSC) was used to analyze the prepared samples, and the melting curves, dissolution behaviors, and enthalpy changes were studied, as shown in Fig. 5. The addition of the graphene aerogel decreased the melting point and increased the freezing point of the paraffin wax to different degrees. As shown in Table 1, the maximum melting point decreased by 1.3 °C and the maximum freezing point increased by 1.7 °C. The relationship between melting point and latent heat of fusion was shown in Fig. 6. With the increased of silver content, the melting point decreased, and so did the latent heat of fusion. This occurred because the graphene aerogel increased the internal thermal conductivity of the paraffin wax, accelerated the heat transfer rate from the outside to the inside, and reduced the overheating phenomenon, thereby reducing the overall melting temperature.^9,17^ The same mechanism was responsible for the change in the solidification process. Increasing the thermal conductivity reduces the undercooling phenomenon, thereby increasing the freezing point temperature.

**Table 1:** Thermal properties of the test materials

	Melting point [°C]	Freezing point [°C]	Enthalpy [J g^−1^]
Paraffin	53.4	46.7	167.4
PRGA	52.5	47.4	165.6
PRGA2A	52.2	47.6	148.5
PRGA6A	52.1	48.4	134.8

The adhesion of the silver particles also affected the thermal conductivity of the composites. That is, it increased thermal conductivity, which reduced the melting point and increased the freezing point. However, PRGA2A and PRGA6A did not have significant effects on the melting and freezing points, which may be due to the small volume of the samples used in the test and the short transmission distance from outside to inside. However, the enthalpy of the composites decreased by 32.6 J g^−1^ owing to the heavier silver particles, which increased the weight of aerogels.

**Fig. 7:**
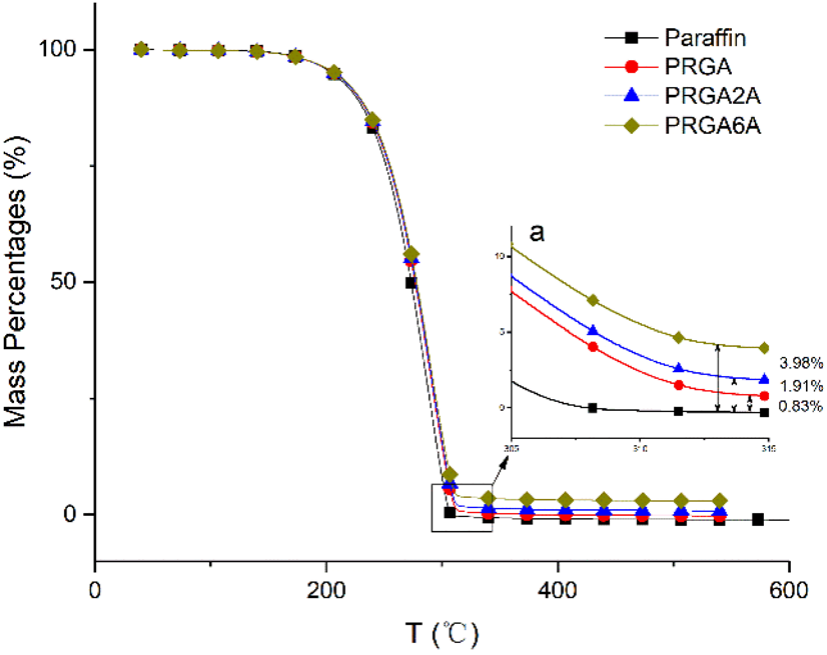
Thermogravimetric curves for the various samples.

Thermogravimetry was used to analyze the thermal decomposition behaviors of the samples, as shown in Fig. 7. The paraffin wax decomposed almost completely at 304 °C. The addition of the graphene aerogel delayed this decomposition to 314 °C, owing to the residual oxidation groups in the graphene sheets, silver oxides, and silver complexes. After decomposition, the residues of the PRGA, PRGA2A, and PRGA6A composites were approximately 0.83%, 1.91%, and 3.98% of the weight of the original specimens, respectively. These residues mainly consisted of residual graphene aerogel and silver particles.

**Fig. 8:**
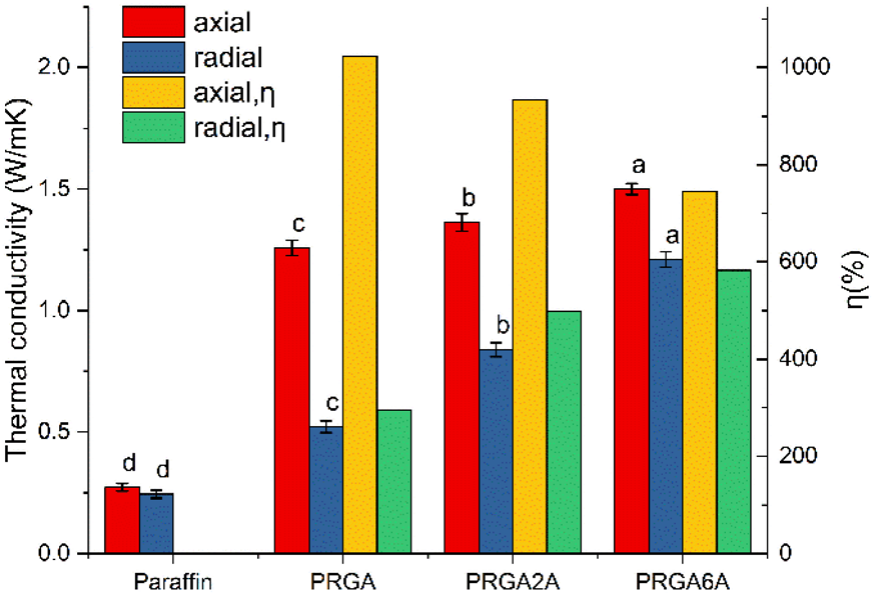
Thermal conductivities of the various samples.

Five samples of each group were prepared, and their thermal conductivity was measured, statistical significance between compared groups was analyzed, as shown in Fig. 8. The addition of the graphene aerogel significantly increased the axial thermal conductivity of the paraffin wax to 1.2452 W m^−1^ K^−1^, which corresponded to an enhancement efficiency of 1023%. By contrast, there was only a slight increase in the radial thermal conductivity of 0.1933 W m^−1^ K^−1^, which corresponded to an enhancement efficiency of 296%. This is because the structure of graphene aerogels was anisotropic, and the graphene sheets were arranged along the axial direction. The thermal conductivity of the graphene sheets was up to 5300 W m^−1^ K^−1^, whereas the interlayer thermal conductivity was much lower than the in-plane thermal conductivity. Therefore, the axial thermal conductivities of the composites were much higher than the radial thermal conductivities. The axial thermal conductivity of PRGA6A was 1.4953 W m^−1^ K^−1^, which was only 0.2501 W m^−1^ K^−1^ higher than that of PRGA. The thermal conductivity enhancement efficiency of PRGA6A was 746% lower than that of PRGA. This is because the heavier silver particles reduced the enhancement efficiency per unit mass of the aerogel. However, the radial thermal conductivity increased substantially to 1.2234 W m^−1^ K^−1^, which is 379% higher than that of pure paraffin wax and 126% higher than that of PRGA. The thermal conductivity enhancement efficiency was up to 582%. Therefore, even though the silver particles reduced the thermal conductivity enhancement efficiency, they greatly improved the radial thermal conductivity by a factor of 4.79 compared to that of pure paraffin wax.

Zhang *et al.*^13^ reported novel and efficient sunlight-driven PCMs based on poly-ethylene glycol (PEG) supported by Ag nanoparticle-functionalized graphene nanosheets (Ag-GNS). The thermal conductivity of this composites was only increased by 49.5–95.3%, which was far lower than the 379% in this paper. Zehri *et al.*^18^ reported a graphene foam (GF) that was produced using chemical vapor deposition (CVD) process and attached to a thermal test chip using sintered silver nanoparticles (Ag NPs). The thermal conductivity of the graphene foam silver composite (GF/Ag) was enhanced by 54%, which was also lower than the enhancement effect in this paper. Fan *et al.*^19^ coated silver/polypyrrole composites on polyurethane PU foam skeletons and the maximum thermal conductivity was 144% higher than that of the pure paraffin, which was far lower than the 379% in this paper. Lv *et al.*^8^ reported a novel reduced graphene oxide aerogel (rGAA) encapsulated PCM by introducing a high aspect ratio of silver nanowires (AgNWs) to a reduced graphene oxide aerogel (rGA). The thermal conductivity was significantly increased to 0.856 W m^−1^ K^−1^, which was 3.21 times that of pure LA and still lower than 4.79 in this paper. In summary, the thermal conductivity enhancement effect of the silver-attached graphene aerogel prepared in this paper is obvious, and it has greater advantages compared with other thermal conductivity enhancement systems of composite silver.

**Fig. 9:**
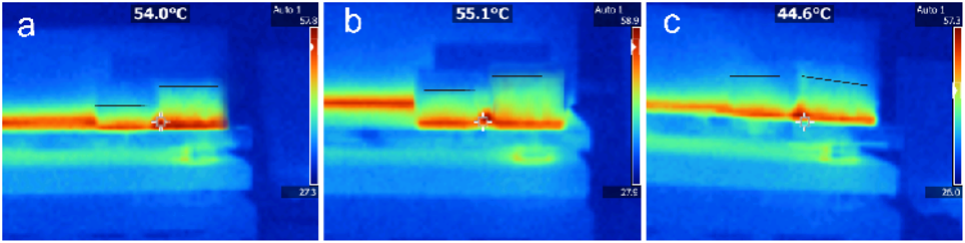
Thermal images of (a) PRGA, (b) PRGA2A, and (c) PRGA6A samples orientated axially (right) and radially (left) on a hot plate.

To further evaluate the heat transfer properties of the composites, their axial and radial heat transfer properties were investigated using a thermal imaging camera. Two square blocks of each composite were prepared. The samples were placed on a heat plate, one orientated axially and the other radially, and heated simultaneously as the temperature distributions were observed, as shown in Fig. 9. In the PRGA samples, the high-temperature region was smaller in the radially orientated sample than the axially orientated sample. By contrast, high-temperature region became closed in the PRGA2A samples, and in the PRGA6A sample, the high-temperature region was approximately the same and in the radially and axially orientated samples. Thus, the radial thermal conductivity of aerogels was obviously improved after silver particles were attached, especially in PRGA6A sample.

## 4. Conclusion

In this study, anisotropic graphene aerogels were prepared *via* the heat flow method, and silver particles ∼120 nm in diameter were attached to the aerogels using a silver mirror reaction. The silver particles improved the radial thermal conductivity of the anisotropic graphene aerogels. The thermal conductivity of pure paraffin wax was 0.2553 W m^−1^ K^−1^. The radial thermal conductivity of PRGA6A prepared with a 6% silver ammonia solution was 1.2234 W m^−1^ K^−1^, which corresponded to a thermal conductivity enhancement efficiency of 582%. The axial thermal conductivity was 1.4953 W m^−1^ K^−1^, which corresponded to a thermal conductivity enhancement efficiency of 746%. These values were significantly higher than those of PRGA without the silver particles, which had radial and axial thermal conductivities of 0.5420 and 1.2452 W m^−1^ K^−1^, respectively. However, the enthalpy of the silver particles was 32.6 J g^−1^ lower than that of pure paraffin wax (167.4 J g^−1^) owing to their large specific gravity. In PRGA6A, the heat transfer in the radial and axial directions was consistent. By contrast, in PRGA, the heat transfer in the radial direction was slower than that in the axial direction.

## Author contributions

Jinhui Huang: conceptualization, investigation, methodology, writing – original draft; Xuejiao Sun: writing – review & editing; Bing Liang: formal analysis; Danyang Zheng: visualization; Ziyao Li: resources; Yonghuang Zhu: project administration, funding acquisition.

## Conflicts of interest

There are no conflicts to declare.

## Supplementary Material
